# Dismantling weight stigma in eating disorder treatment: Next steps for the field

**DOI:** 10.3389/fpsyt.2023.1157594

**Published:** 2023-04-11

**Authors:** Mindy L. McEntee, Samantha R. Philip, Sean M. Phelan

**Affiliations:** ^1^College of Health Solutions, Arizona State University, Phoenix, AZ, United States; ^2^Department of Psychological and Brain Sciences, Texas A&M University, College Station, TX, United States; ^3^Division of Health Care Delivery Research, Robert D. and Patricia E. Kern Center for the Science of Health Care Delivery, Mayo Clinic, Rochester, MN, United States

**Keywords:** weight stigma, weight bias, eating disorders, eating disorder treatment, weight-inclusive care, binge eating, atypical anorexia, health equity

## Abstract

The authors posit current guidelines and treatment for eating disorders (EDs) fail to adequately address, and often perpetuate, weight stigma. The social devaluation and denigration of higher-weight individuals cuts across nearly every life domain and is associated with negative physiological and psychosocial outcomes, mirroring the harms attributed to weight itself. Maintaining focus on weight in ED treatment can intensify weight stigma among patients and providers, leading to increased internalization, shame, and poorer health outcomes. Stigma has been recognized as a fundamental cause of health inequities. With no clear evidence that the proposed mechanisms of ED treatment effectively address internalized weight bias and its association with disordered eating behavior, it is not hard to imagine that providers’ perpetuation of weight bias, however unintentional, may be a key contributor to the suboptimal response to ED treatment. Several reported examples of weight stigma in ED treatment are discussed to illustrate the pervasiveness and insidiousness of this problem. The authors contend weight management inherently perpetuates weight stigma and outline steps for researchers and providers to promote weight-inclusive care (targeting health behavior change rather than weight itself) as an alternative approach capable of addressing some of the many social injustices in the history of this field.

## 1. Introduction

Eating disorder (ED) treatment has been developed within the context of a weight-centric paradigm of medicine that views higher body weight as unhealthy and asserts that *weight loss* can improve health. However, this approach vastly oversimplifies the complexity of body weight and fails to consider empirical evidence contradicting its underlying assumptions. Critiques of the weight-centric health paradigm have been discussed in detail (1–4), yet the evidence supporting alternative models of care have not informed ED treatment. Central to these alternative models are three tenets: (1) there is no *causal* evidence supporting the link between higher body weight and disease or premature death (see the critiques cited above), (2) BMI is not empirically supported as a measure of an individual’s health (5), and (3) interventions targeting *health behaviors* have shown to improve health independent of weight outcomes.

Healthcare that explicitly focuses on weight can intensify stigma among both patients and providers, leading to increased internalization, shame, and poorer health outcomes (6–9). Reliance on BMI and the weight of those in ED treatment further conflates weight with health, particularly among ED care providers who may harbor weight bias (10) and in the absence of intentional efforts to address fat phobia and social injustice related to weight stigma. As stigma has been recognized as a fundamental cause of health inequities (11), this article reviews the adverse effects of weight stigma in healthcare with a focus on current practices that may be contributing the development, maintenance, and limited success of ED treatment. The purpose of this article is to encourage individuals in the field to re-examine the evidence underlying current assumptions and clinical practices that may be perpetuating weight stigma and propose some suggestions that effectively shift the target from a focus on weight to more readily modifiable health behaviors to promote greater equity in care.

## 2. Weight stigma definition and manifestations

Weight stigma is the social denigration of individuals on the basis of their weight and/or body shape (12). One component of weight stigma is weight bias, the negative attitudes toward and assumptions about individuals because of their weight. Larger-bodied individuals often face negative stereotypes, such as “lazy,” or “incompetent,” rooted in the assumption that their body size/shape is indicative of moral failing (13, 14). Weight-based stereotypes are often internalized and used to self-denigrate in a process known as internalized weight stigma (15).

Persisting as a socially acceptable and pervasive form of prejudice and discrimination (16, 17), weight stigma presents across settings and emerges on individual, interpersonal, institutional, and societal levels. This article focuses primarily on individuals with larger bodies in ED treatment, acknowledging they are more likely to encounter stigma as a byproduct of fat phobic societal ideals, attributions of weight to personal responsibility, and the inherent visibility of body size/shape (relative to other sources of stigma that may be more easily concealed). However, it is important to remember that weight stigma and its effects occur across the weight spectrum (18). Weight stigma is complex in that it is associated with poorer psychosocial functioning and well-being in a transdiagnostic manner (19) while also intersecting with other forms of stigma, including stigma surrounding eating disorders. In the latter case, both stigmas often emerge from attributions of personal responsibility which function to further stigmatize these individuals (20).

### 2.1. Weight stigma and health outcomes

Weight stigma is associated with a host of adverse physical and psychological outcomes, yet remains overshadowed by the volume of literature equating weight and health (3). In a 2018 systematic review, researchers found that after controlling for weight status, weight stigma predicted elevation in biomarkers associated with hypothalamic–pituitary–adrenal axis reactivity, systemic inflammation, and type 2 diabetes mellitus (9). Two large, nationally representative, longitudinal studies found that experiences of weight discrimination were associated with a 60% increased risk of mortality *not explained by BMI or other clinical and behavioral risk factors* (21). The stress of weight stigma is also deleterious to mental wellbeing: accounting for weight status, stigma has been associated with greater risk of mood, anxiety, and substance use disorders, elevated suicidality, heightened interpersonal sensitivity, social isolation, lower self-esteem, body dissatisfaction, and risky weight control behaviors (9, 22, 23).

## 3. Importance of stigma to ED pathology and treatment

Commonly used self-report measures distinguish between *experienced* (e.g., Stigmatizing Situations Inventory) and *internalized* weight stigma (e.g., Weight Bias Internalization Scale, Weight Self-Stigma Questionnaire). Both the experience and internalization of weight stigma have been established as critical risk factors in the development and maintenance of eating pathology (24–26). Internalized weight stigma has been identified as a key moderator of intervention outcomes (27) and as a mediator between the experience of weight stigma and ED symptomatology (28). Implicit anti-fat/pro-thin attitudes, which develop over time with increased exposure to thin-ideal messages, have also been shown to be independently predictive of EDs (29).

While the literature indicates the importance of weight stigma as a treatment target in ED care, common treatment modalities devote little (if any) attention to anti-fatness as a social injustice and fail to provide tools to identify, validate, and cope with anti-fat prejudice or its internalization. Although “fear of fatness” is recognized in the transdiagnostic theory of EDs, this construct is not an explicit treatment target in the field’s gold standard treatment, Cognitive Behavioral Therapy (CBT, 30). Further, there is insufficient evidence that CBT is effectively offsetting the link between internalized weight stigma and disordered eating (31). Providers’ own weight bias may explain this oversight in treatment.

## 4. Provider weight bias

Healthcare providers are a primary source of weight stigma (3, 8, 32, 33), which can lead to spending less time with and being more critical of higher-weight patients (34), along with greater reluctance to provide routine medical care and education (35). It’s also associated with poorer patient-provider communication, loss of trust and rapport, and decreased patient engagement in care, as indicated by reduced adherence to treatment recommendations and greater avoidance of healthcare (7). While most literature in this area focuses on physicians, research suggests that mental healthcare providers also hold high levels of weight bias. For example, in one study, a majority of ED practitioners reported observing colleagues in their field making negative comments about higher-weight patients, nearly half believe that ED practitioners hold negative stereotypes of higher-weight patients, and one-third reported that their colleagues harbor weight bias and are uncomfortable treating patients with “obesity” (10). A study among higher-weight individuals with a history of an ED corroborates these findings; nearly half of the sample reported that providers recommended dieting and 40% reported that their providers encouraged disordered eating behaviors or attitudes in service of weight loss (36).

## 5. Current ED treatment success

Despite eating disorders being among the most lethal forms of mental illness (37), there is limited evidence supporting the overall efficacy of current ED treatments (38–41). In the following sections, we present ways in which weight bias and stigma exposure may contribute to suboptimal response/recovery rates.

## 6. Impact of the weight-centric paradigm on ED development

Mounting evidence demonstrates that Body Mass Index (BMI) is not the strong indicator of health it was once believed to be. Large epidemiological studies indicate comparable mortality risk for those in the “grade 1 obesity” and “normal” BMI categories (42), with those in the “overweight” category at lowest risk, suggesting the link between body weight and mortality has been overstated. Secondly, BMI does not account for gender or ethnicity and was never intended for use in assessing an *individual’s* health (5, 43). Finally, evidence does not support the claim that higher BMI *causes* poor health. While BMI is *correlated* with certain health conditions, other factors, including weight stigma, weight cycling, and health behaviors, may be more likely to explain the observed associations between BMI and health conditions (8, 44).

Numerous studies have shown that behavioral weight loss interventions can lead to improved cardiometabolic health (45). The issue with this evidence is that there are two mechanisms of action: weight loss itself, and the behaviors that led to weight loss. It is challenging to separate the two mechanisms since they co-occur frequently in these studies. There is strong evidence that behaviors improve health, as demonstrated in studies where health was improved without weight loss (46–48). Fewer studies have examined the impacts of weight loss without behavior change. One example is a 2004 study of health impacts of abdominal liposuction, which found no correlation between weight loss (from removal of adipose tissue) and health benefits in the absence of behavior change (49). A second example is a 2021 study that found weight loss post-bariatric surgery was not correlated with improved cardiometabolic health (50). While these examples do not fully discredit the hypothesis that weight in and of itself predicts health improvement, they do support the hypothesis that behaviors which led to weight loss in much of the extant literature is doing the heavy lifting in improving people’s health.

In addition to the lack of evidence that intentional weight loss produces improved health outcomes independent of behavior change (46), decades of research indicate that the majority of individuals are unable to effectively reduce their body size long term (51). *Weight loss, then, is neither a prerequisite for improved health nor an appropriate target for treatment*. Despite this, providers routinely recommend weight loss to patients with a BMI over 30 (52). Intentional pursuit of weight loss often leads to restrictive and compensatory behaviors, when we know dieting (53) and attempts to control one’s weight are associated with greater risk for developing EDs (54). As astutely noted by Debora Burgard, fat-positive clinician and activist (55), “we find it hypocritical to prescribe practices for heavier people that we would diagnose as eating disordered in thin ones.” Indeed, higher-weight individuals are more likely to experience delays in ED diagnosis and treatment and have, on average, lost more weight prior to ED treatment (56, 57), worsening the overall ED prognosis (58). Higher-weight patients may be more prone to delaying or avoiding treatment due to internalized stigma and past negative experiences with physicians (59). Primary care physicians, who are well-positioned to serve as the first line of defense in the screening of EDs (60), may instead inadvertently reinforce a patient’s ED by commenting on their weight or praising weight loss.

## 7. Weight stigma in ED treatment

Weight-centric practices impact case conceptualization and ED treatment. Listed below are some examples of weight stigma reported by clinical providers and former patients in ED treatment in the US. While much of this research has focused on Western countries, its adverse effects are an issue globally (61–65).

### 7.1. Reliance on unsupported weight-based classifications

Reference to BMI or related terminology such as “overweight” or “obese” reinforce arbitrary classifications that are not empirically supported as a measure of individual health. Such terms pathologize larger body size and perpetuate weight stigma, yet are ingrained in ED providers’ vernacular and public health messaging (e.g., “the obesity epidemic,” “the war on obesity”). BMI has historically been used as a diagnostic criterion for Anorexia Nervosa (AN), which led to higher-weight individuals being labeled with “atypical anorexia nervosa” (AAN) despite being the more common presentation of AN. AAN is often perceived as less severe (66), which may prevent higher-weight individuals with severe restrictive pathology from receiving immediate and life-saving services (56). BMI does not necessarily reflect problematic behaviors utilized to reach or maintain a current weight, nor does it adequately contextualize an individual’s current weight with respect to their developmental history. To this point, recent research has shown BMI-based severity ratings for AN in the DSM-5 fail to accurately identify biological markers of medical concern (67). Alterative metrics, such as overvaluation of weight/shape (67) and weight history (68) have been shown to be better indicators of malnutrition for AN. The authors take this a step further with the suggestion that providers recommending “weight management” or simultaneously treating “EDs and obesity” perpetuate weight stigma by maintaining an explicit focus on weight (rather than health behaviors) as the target for treatment.

### 7.2. Provider diagnosis and treatment recommendations

Data consistently suggests that individuals with higher body weight are less likely to be diagnosed with an eating disorder and experience a longer delay before receiving treatment (57). Providers are also more likely to perceive eating disorders as less severe and recommend less intensive treatments for larger bodied individuals (66, 67). Observed examples of this in practice include prescribing lower calorie (more restrictive) meal plans to larger bodied patients or offering combined treatment for Binge Eating Disorder and “Obesity.” Such treatment conflates recovery from an ED with goals and activities that may have contributed to its development.

### 7.3. Weigh-ins without adequate rationale or communication

Weight monitoring is frequently incorporated into ED treatment, with variability regarding the frequency, timing, and other parameters surrounding weigh-ins. Mandated weighing can reinforce the false notion that weight is a stronger indicator of health than dietary habits, physical activity, and other health behaviors. To date, no dismantling studies have identified the utility of measuring weight in ED treatment.

Weight status is also problematically used to mark treatment “progress” and “recovery” with EDs. Although weight restoration is a crucial component of treatment for anorexia nervosa, there is no empirical consensus in the field for setting target weights (69). As such, many providers default to use of BMI to define one’s “healthy weight,” which fails to consider individual differences in body composition or stunted height due to malnourishment and can lead to recovery weights being set too low. For those whose baseline weight was higher than “average” prior to admission, prescribed “healthy weights” may represent significant weight suppression, or at least expect patients to prioritize weight gain as healthy and important right before reversing that expectation and calling “excessive” weight gain unhealthy. This common practice may be confusing to patients as they work to reset and redefine their relationship with food. From a harm reduction standpoint, if weigh-ins are required, it should be clearly communicated to the patient why they are necessary, how they fit within a more comprehensive treatment plan, and how weight stigma will be addressed. Doing this once at the start of treatment is likely insufficient.

### 7.4. Treatment (unintentionally) reinforcing the fat-phobic attitudes

Providers have responded to patient concerns of loss of control by giving reassurance they will “not allow them to go overboard.” Whether intended as a means of building rapport or increasing patient buy-in, this sentiment reinforces fat phobia and perpetuates the idea that a higher weight symbolizes gluttony or unhealthiness. Efforts to reframe language within CBT are another example: using phrases such as “fat is not a feeling” and “you are not fat, you *have* fat” fail to challenge the underlying assumption that fat = bad while minimizing the life experiences of those in larger bodies. When fear of fatness is incorporated into treatment, it is often addressed only in the context of reality testing distorted cognitions rather than questioning the weight-centric practice of equating weight and fat with health. As such, body image is often addressed in ways that reinforce fat-phobic attitudes rather than challenge weight stigma. Some types of treatment may lend themselves to focusing on health behaviors rather than weight (e.g., weight-inclusive care) more readily than others (e.g., Acceptance and Commitment Therapy), although care needs to be taken to ensure treatment elements aren’t functioning in ways that unintentionally perpetuate stigma.

### 7.5. Discontinuity of care/lack of provider communication

Patients with eating disorders often see multiple providers for different aspects of their healthcare. Lack of care coordination or provider communication can undermine treatment if providers are not working toward a shared treatment plan. For instance, a primary care provider may make weight loss recommendations or congratulate a patient with atypical anorexia nervosa on recent weight loss if they are not in frequent contact with the ED treatment team or have not recently consulted the patient’s chart.

### 7.6. Provider endorsement of negative stereotypes

Providers have been overheard making judgments about the quality, quantity, and/or type of food patients select, particularly in outpatient settings where this involves patient choice (e.g., “they are just eating junk”). Providers have also been overheard making assumptions about a patient’s motivation or effort in treatment (e.g., “they aren’t even trying anymore”) and casual comments that impart judgment based on body weight, shape, or size (e.g., “I’ve been so lazy lately, [the way] my pants [fit] are telling me I need to get back to running”). Even if such comments are directed at coworkers rather than patients, they perpetuate a culture of weight stigma and are more likely to cause harm than help. Interventions to reduce weight bias among healthcare providers have targeted education on the controllability of weight, increased empathy, self-reflection/awareness of personal biases, cognitive dissonance, and/or contrasting negative personal attitudes with positive social consensus with little evidence of effect (70, 71). Notably, this area of research has focused on evaluating immediate changes in provider attitudes (rather than provider behavior or patient outcomes) and often occurs in the context of weight-centric care, thereby failing to address sociocultural factors affecting provider behavior. The complexity of this issue suggests the need for multilevel interventions to shift from a weight-centric to weight-inclusive paradigm and evaluate observable changes in provider behavior, clinic policies and workflows, and patient outcomes.

## 8. Discussion

To date, there has been little effort to directly address weight stigma in the treatment of EDs. While data has clearly shown iatrogenic effects of weight-centric care, proposed guidelines for treating EDs in higher-weight individuals (72) skirt around the issue, noting “obesity management” falls outside the scope of their clinical recommendations. Recent guidelines from the American Academy of Pediatrics (73) similarly perpetuate the unsupported assumption that weight itself (“obesity”) is problematic and a necessary target for clinical intervention. The fact remains that weight is not synonymous with health. Given the limited evidence supporting use of BMI at an individual level and data demonstrating health behavior change can improve health irrespective of weight, we contend that efforts to address weight stigma while promoting weight management are ineffective and perpetuate the stigma they have identified as problematic, particularly among individuals with EDs in larger bodies.

There is empirical evidence supporting weight inclusive care as an alternative to the prevailing weight-centric paradigm in medicine (47, 74, 75), yet there remains little guidance on how to apply this to EDs. The recommendations discussed below and in Table 1 reflect our vision for moving the field forward by addressing weight stigma and other historical biases in the field (e.g., racism, classism, sexism) to improve treatment outcomes and health equity.

**Table 1:** Recommendations to foster a weight-inclusive paradigm in ED treatment and research.

Acknowledge the influence of weight stigma on current ED treatment.
Understand and communicate with patients, policy-makers, and other providers that weight is not synonymous with health and, accordingly, weight is inadequate as a criterion for ED diagnosis or recovery
Re-examine the rationale for and unintended effects of weighing in ED treatment
**Shift from weight-centric to weight-inclusive care practices and philosophy.**
Stop recommending or engaging clients in weight loss or weight management
Focus on modifiable health behaviors (regardless of weight status, with respect for an individual’s history), for the purpose of improving quality of life rather than moralization of health and health behavior
Ensure treatment plans and goals function to increase a client’s flexibility in behavioral responses (rather than “managing” their weight)
Work to increase client autonomy, access to care, and social justice across the weight spectrum (76) by explicitly addressing weight stigma in treatment
**Increase provider education and competency.**
Promote widespread dissemination of weight-inclusive care as an empirically-supported alternative to the weight-centric medical paradigm in training programs for anyone working with clients to improve health
Encourage use of functional behavioral assessments and other direct health measures instead of reliance on weight/BMI
Utilize shared treatment plans to enhance provider communication and continuity of care
Build in mechanisms to increase accountability for provider weight bias, prioritizing meaningful behavior change and structural interventions to reduce stigmatizing behaviors (versus unrealistic expectations of trying to “eliminate” personal bias)
**Prioritize addressing gaps in research and clinical care.**
Increase clinical training and research with diverse populations to better understand variability in symptom presentation and how to assess ED severity across the weight spectrum
Incorporate and evaluate the effects of addressing weight stigma as an explicit part of treatment, particularly at areas in which weight is interconnected with other areas of historical bias and social marginalization (race, gender, sexual orientation, class, ability, etc.). Such research should utilize mixed methods to better contextualize findings
Policies should be regularly reviewed and responsive to the data, including increased funding for weight-inclusive care (rather than additional weight loss interventions)

ED treatment does not exist in a vacuum; patients and providers are both impacted by the larger societal system(s) in which they are embedded. The intent of this article is not to point fingers or induce shame, but to prompt reflection on the assumptions and practices that have emerged from a weight-centric paradigm and lack causal supporting data. There is a clear need to address provider weight bias and competency in treating EDs. Principally, this includes a greater understanding of the lack of causal evidence equating weight with health in contrast to the efficacy of health behavior change. Efforts to improve care for EDs must move past treating the field as an isolated specialty. Widespread dissemination of weight-inclusive care would include all professionals working with clients to improve health and may be most effective and efficient if introduced in training programs with supportive supervisors who model weight-inclusive care practices. Success in this area is likely to require multilevel and structural interventions. Top-down administrative approaches may facilitate change processes, but are more likely to be successful when paired with bottom-up strategies, such as provider champions advocating for weight-inclusive care from within an organization. We need leaders in the ED field to advocate for this as a priority area and coordinate action at multiple levels.

As the weight-centric paradigm is embedded within a broader sociocultural preference for leanness over fatness, a message reinforced through multiple communication channels and across domains (e.g., healthcare, media, education, the workplace), we can anticipate resistance to change. It remains critical that we acknowledge the contextual history and the biases that have shaped the field and actively work toward more equitable treatment. This includes the use of a broader systems approach to better understand the complex interplay between personal characteristics and an individual’s environment, which may identify additional leverage points for intervention. Awareness of the issue is not sufficient. We must be held accountable for the potential harm of our actions and actively intervene at multiple levels if we hope to improve health equity and ED treatment.

## Data availability statement

The original contributions presented in the study are included in the article/supplementary material, further inquiries can be directed to the corresponding author.

## Author contributions

MM, SRP, and SMP contributed to the writing and revision of this manuscript and approved of the submitted version. All authors contributed to the article and approved the submitted version.

## Conflict of interest

The authors declare that the research was conducted in the absence of any commercial or financial relationships that could be construed as a potential conflict of interest.

## Publisher’s note

All claims expressed in this article are solely those of the authors and do not necessarily represent those of their affiliated organizations, or those of the publisher, the editors and the reviewers. Any product that may be evaluated in this article, or claim that may be made by its manufacturer, is not guaranteed or endorsed by the publisher.
